# Radiation Exposure and Local Diagnostic Reference Levels During Endovascular Treatment of Cerebral Arteriovenous Malformations and Dural Arteriovenous Fistulas

**DOI:** 10.3390/biomedicines14061251

**Published:** 2026-05-30

**Authors:** Mariusz Stanisław Sowa, Joanna Sowa, Maciej Budzanowski

**Affiliations:** 1 Department of Neurology and Neurosurgery, Faculty of Medicine, Collegium Medicum, University of Warmia and Mazury in Olsztyn, Aleja Warszawska 30, 10-082 Olsztyn, Poland; neurochirurgia@uwm.edu.pl; 2 Interventional Dosimetry Laboratory at the Department of Neurosurgery, University Hospital in Olsztyn Aleja Warszawska 30, 10-082 Olsztyn, Poland; 3 Department of Radiation Physics and Dosimetry, Institute of Nuclear Phisics, Polish Academy of Sciences, 31-342 Kraków, Poland; maciej.budzanowski@ifj.edu.pl

**Keywords:** DRL, diagnostic reference levels, AVM treatment, Histoacryl, AVF treatment, radiation dose optimization

## Abstract

**Background/Objectives:** Endovascular treatment of cerebral arteriovenous malformations (AVMs) and arteriovenous fistulas (AVFs) is associated with substantial radiation exposure due to procedural complexity and repeated angiographic acquisitions. This study evaluates radiation exposure during AVM and AVF embolization and establishes local diagnostic reference levels (DRLs). **Methods:** A single-center retrospective dose audit was conducted, encompassing 114 endovascular procedures performed using a low-dose workflow. Radiation exposure was quantified using dose area product (DAP), reference air kerma (K_a,r_), fluoroscopy time (FT), and the number of digital subtraction angiography (DSA) frames per procedure. Median values were defined as the median (P50), and local DRLs as the 75th percentile (P75). Comparative analyses were conducted between AVM and AVF procedures, between male and female patients, and within selected AVM subgroups. **Results:** The analysis comprised 86 AVM procedures and 28 AVF procedures. For AVMs, the local DRLs (P75) were 28.9 Gy·cm^2^ for DAP, 400 mGy for K_a,r_, 310 DSA frames per procedure, and 1619 s for FT. For AVFs, the respective values were 47.3 Gy·cm^2^, 465 mGy, 478 DSA frames, and 1820 s. No statistically significant differences were identified between female and male patients. However, AVF procedures demonstrated significantly higher radiation exposure than AVM procedures for all parameters except FT. Within the AVM subgroup, no significant differences were observed between single-stage and other AVM procedures or between female and male patients. **Conclusions:** AVM and AVF embolization procedures are dose-intensive neuroendovascular interventions. Establishing local DRLs for AVM and AVF may enhance radiation monitoring and facilitate procedure-specific dose optimization.

## 1. Introduction

Intracranial vascular malformations comprise a heterogeneous spectrum of lesions, each with distinct angioarchitecture, natural history, and clinical significance. Brain arteriovenous malformations (bAVMs) are characterized by a nidus that allows arterial blood to shunt directly into the venous circulation, bypassing the capillary network. Although uncommon at the population level, bAVMs constitute a significant cause of intracranial hemorrhage and neurological morbidity, particularly among younger adults [1].

The clinical course of bAVMs is variable. Some patients present with hemorrhage, while others develop seizures, headache, or focal neurological deficits. The risk of hemorrhage is influenced by a combination of clinical and imaging factors, such as prior rupture, deep location, and venous drainage pattern. These factors inform individualized risk–benefit assessments when determining an appropriate treatment strategy [2].

A distinct subset of intracranial arteriovenous shunts includes arteriovenous fistulas (AVFs), particularly dural AVFs (dAVFs) [3,4,5]. These lesions are characterized by pathological shunts between meningeal arterial feeders and dural venous sinuses or dural veins, typically in the absence of a parenchymal nidus. DAVFs are generally regarded as acquired lesions, and their clinical prognosis is primarily determined by venous drainage patterns, such as cortical venous reflux, as classified by the Borden and Cognard systems [6]. In this multimodal context, radiation exposure should not be considered as a single-procedure issue only. Patients with AVM or AVF may undergo diagnostic angiography, therapeutic embolization, surgical or radiosurgical treatment, and serial angiographic follow-up.

AVM and AVF management may involve single-modality or multimodal approaches, including endovascular embolization, microsurgical resection, and stereotactic radiosurgery (SRS). In many cases of dAVF, endovascular therapy is considered the first-line treatment. Conversely, embolization is often applied in bAVM either as a definitive therapy in selected cases or as part of combined strategies, such as pre-operative intervention [6,7,8,9].

Neuroendovascular interventions are inherently guided by fluoroscopy and DSA, which expose patients to ionizing radiation. Effective patient radiation protection necessitates minimizing dose indicators [10] while maintaining procedural safety and adequate imaging quality, especially during complex or prolonged interventions that carry a higher risk of tissue reactions and increased cumulative lifetime exposure [11,12].

Diagnostic reference levels (DRLs) serve as a practical optimization tool. These distribution-based “investigation levels” are designed to prompt local review when typical dose indicators are consistently high or implausibly low, which may compromise image quality. DRLs do not represent individual patient dose limits [13].

This study aimed to establish local DRLs for endovascular embolization of bAVM, AVF, and dAVF at the University Hospital in Olsztyn. This was achieved through a retrospective analysis of procedures conducted between July 2018 and March 2026. Distributions of dose indicators, including patient dose air kerma product (PKA), dose area product (DAP), reference air kerma (K_a,r_), fluoroscopy time (FT), and technical parameters, were evaluated in relation to patient and lesion characteristics [14]. The study was designed as a procedural dose audit and DRL analysis, not as a clinical outcomes study or a controlled comparison between low-dose and conventional angiographic protocols.

## 2. Materials and Methods

A retrospective dose audit study of neurointerventional procedures was conducted at a single center from 2018 to 2024. The primary objective was to characterize dose indicator distributions and establish local DRLs for specific clinical categories of endovascular treatment of brain AVMs and intracranial AVF/dAVF. The study adhered to ethical standards and data confidentiality requirements. Only retrospectively obtained clinical and procedural data were analyzed, with all records anonymized. In accordance with applicable regulations, review and approval by the Bioethics Committee of the Faculty of Medicine, Collegium Medicum, University of Warmia and Mazury, were not required. The study population comprised 63 women and 51 men, aged 19 to 86 years, with a mean age of 55.4 years (Table 1). Embolization was undertaken in all AVM and AVF cases using Histoacryl and embolization coils.

**Table 1:** Characteristics of cerebral arteriovenous malformation and arteriovenous fistula procedures. The total number of procedures exceeds the number of patients because some individuals underwent multiple interventions. Values are mean ± SD.

Parameter	Arteriovenous Fistula			Arteriovenous Malformation			All
Sex	Female	Male	Total	Female	Male	Total	
Number of procedures	20	8	28	43	43	86	114
Age (years)	59.1 ± 16.1	57.1 ± 16.4	58.6 ± 15.9	43.5 ± 14.9	52.0 ± 15.1	47.8 ± 15.5	50.4 ± 21.2
Height (m)	1.64 ± 4.2	1.76 ± 4.8	1.67 ± 6.8	1.65 ± 4.4	1.70 ± 2.56	1.69 ± 1.83	1.68 ± 1.64
Body weight (kg)	73.8 ± 14.1	99.4 ± 35.8	81.0 ± 35.8	65.7 ± 16.2	82.7 ± 19.9	73.4 ± 19.8	75.3 ± 21.2

The analysis was conducted at the procedure level to account for patients who received staged or repeated treatments. Detailed characteristics of the AVF and AVM procedures are provided in Table 2 and Table 3.

**Table 2:** Baseline characteristics of patients and distribution of dural arteriovenous fistula (dAVF) procedures by anatomical location.

Parameter	Number of Procedures
Number of patients	18
Total number of procedures	28
Males/females	8/10
Patients with one procedure	13
Patients with two procedures	1
Patients with three procedures	3
Patients with four procedures	1
Tentorium cerebelli AVF	12
Middle cranial fossa AVF	8
Anterior cranial fossa AVF	5
Posterior cranial fossa AVF	2
Iatrogenic carotid–vertebral fistula	1

**Table 3:** Characteristics of arteriovenous malformation procedures included in the study.

Parameter	Number of Procedures
Number of patients	54
Total number of procedures	86
Patients with one procedure	35
Patients with two procedures	9
Patients with three procedures	7
Patients with four procedures	3
Spetzler–Martin grade I	4
Spetzler–Martin grade II	13
Spetzler–Martin grade III	9
Spetzler–Martin grade IV	3
Spetzler–Martin grade V	3
Skull base AVM	6
Nasal cavity AVM	12
Spinal canal lesion with fistula	1
Orbital lesion	1
Sturge-Weber syndrome-related lesion	1
Lesion initially diagnosed as hemangioblastoma	1

All procedure sessions that met the clinical indication definition and satisfied the minimum dosimetric data completeness criteria were included in the analysis to minimize selection bias. Consecutive cases were included, while exclusions were limited to sessions where exposure could not be reliably reconstructed, such as those with missing dose reports or cases that did not align with the predefined clinical category, such as diagnostic angiography without treatment. Dosimetric data were extracted from system dose reports, and clinical variables from medical records. The cohort should be interpreted as a pragmatic procedural dose-audit cohort rather than as a natural-history cohort restricted exclusively to classical parenchymal brain AVMs. Non-classical arteriovenous shunting lesions, including skull-base, nasal cavity, orbital, spinal, and syndromic lesions, were retained because they required therapeutic endovascular embolization and comparable angiographic guidance, acquisition planning, and dose monitoring. This inclusion strategy reflects the clinical and anatomical spectrum of arteriovenous shunting lesions managed in highly specialized neurointerventional center. However, because of the anatomical and pathological heterogeneity of the present cohort and the limited size of specific lesion subgroups, subgroup analyses should be regarded as exploratory.

**Figure 1:**
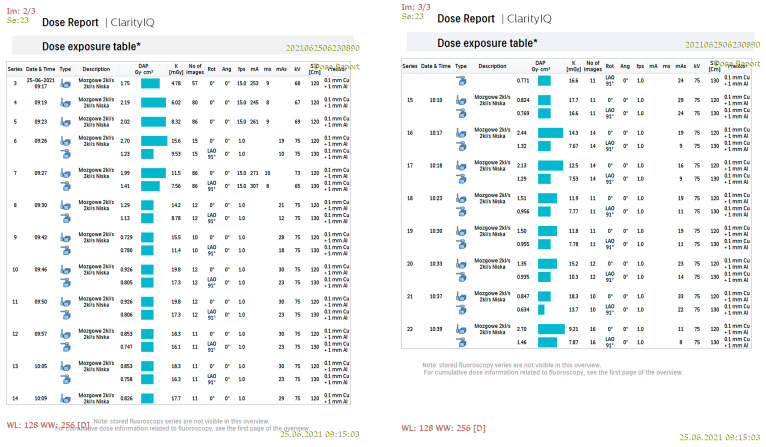

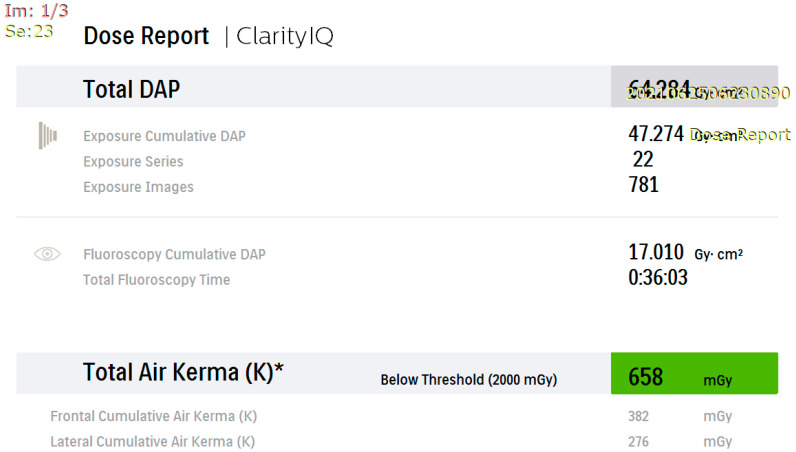
Example of a dose report generated using a biplane angiographic system during the treatment of a left-sided tentorial dural arteriovenous fistula (dAVF) in Patient X. * Air kerma is reported at the interventional reference point (IRP), located 15 cm from the isocenter toward the X-ray tube. The dose report includes the entry: “mózgowe 2 kL/s, 2 kL/s niska” (“Cerebral 2 fps, 2 fps Low”).

**Figure 2:**
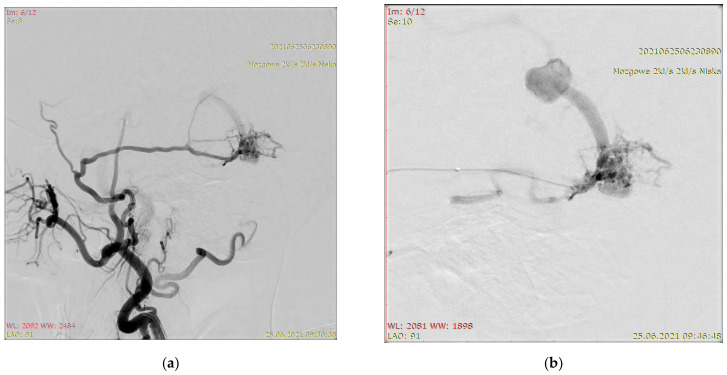

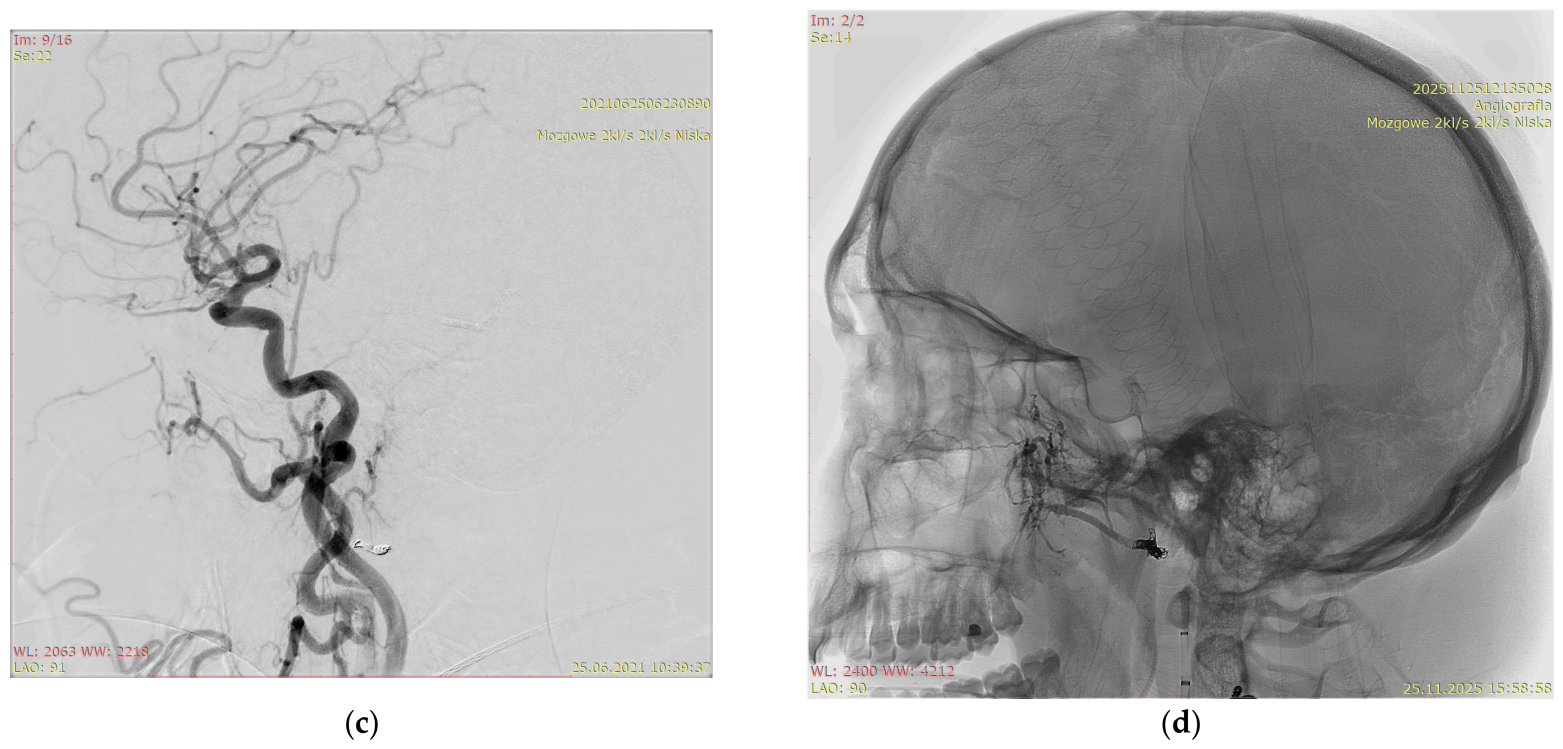
Left-sided tentorial dural arteriovenous fistula (dAVF) in Patient X. (**a**) Pre-treatment image of the dAVF, (**b**) microarteriography of the lesion, (**c**) post-treatment vascular image, (**d**) X-ray image showing the embolic material. The term “mózgowe 2 kL/s, 2 kL/s niska” translates as “Cerebral 2 fps, 2 fps Low”.

**Figure 3:**
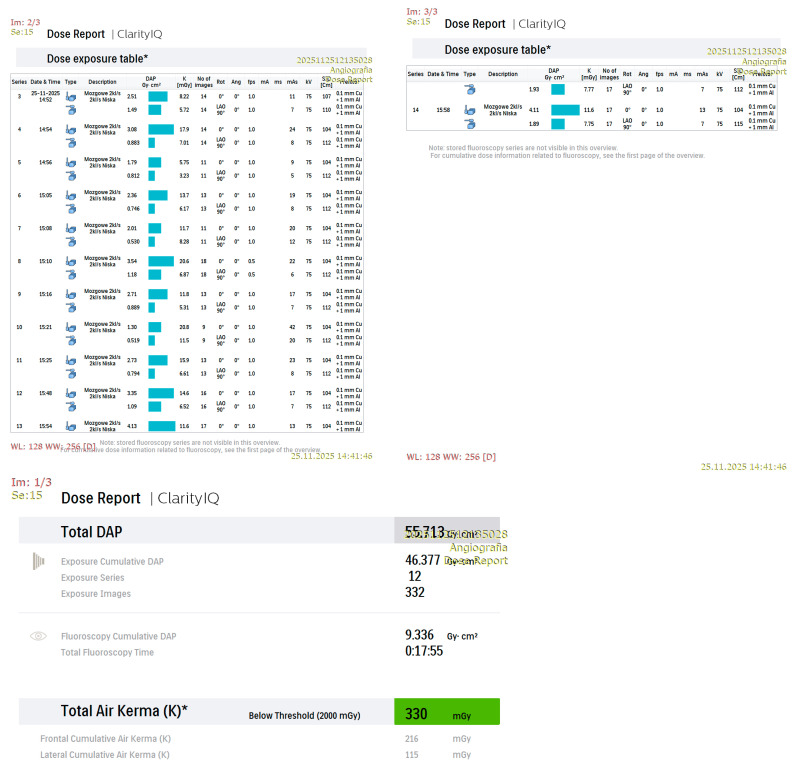
Representative example of a dose report generated using a biplane angiographic system during treatment of a skull-base arteriovenous malformation (AVM) in Patient Y. * Air kerma is reported at the interventional reference point (IRP), located 15 cm from the isocenter toward the X-ray tube. The dose report contains the entry: “mózgowe 2 kL/s, 2 kL/s niska” (“Cerebral 2 fps, 2 fps Low”).

**Figure 4:**
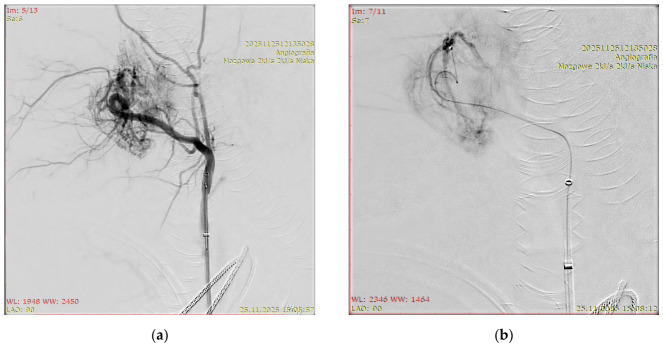

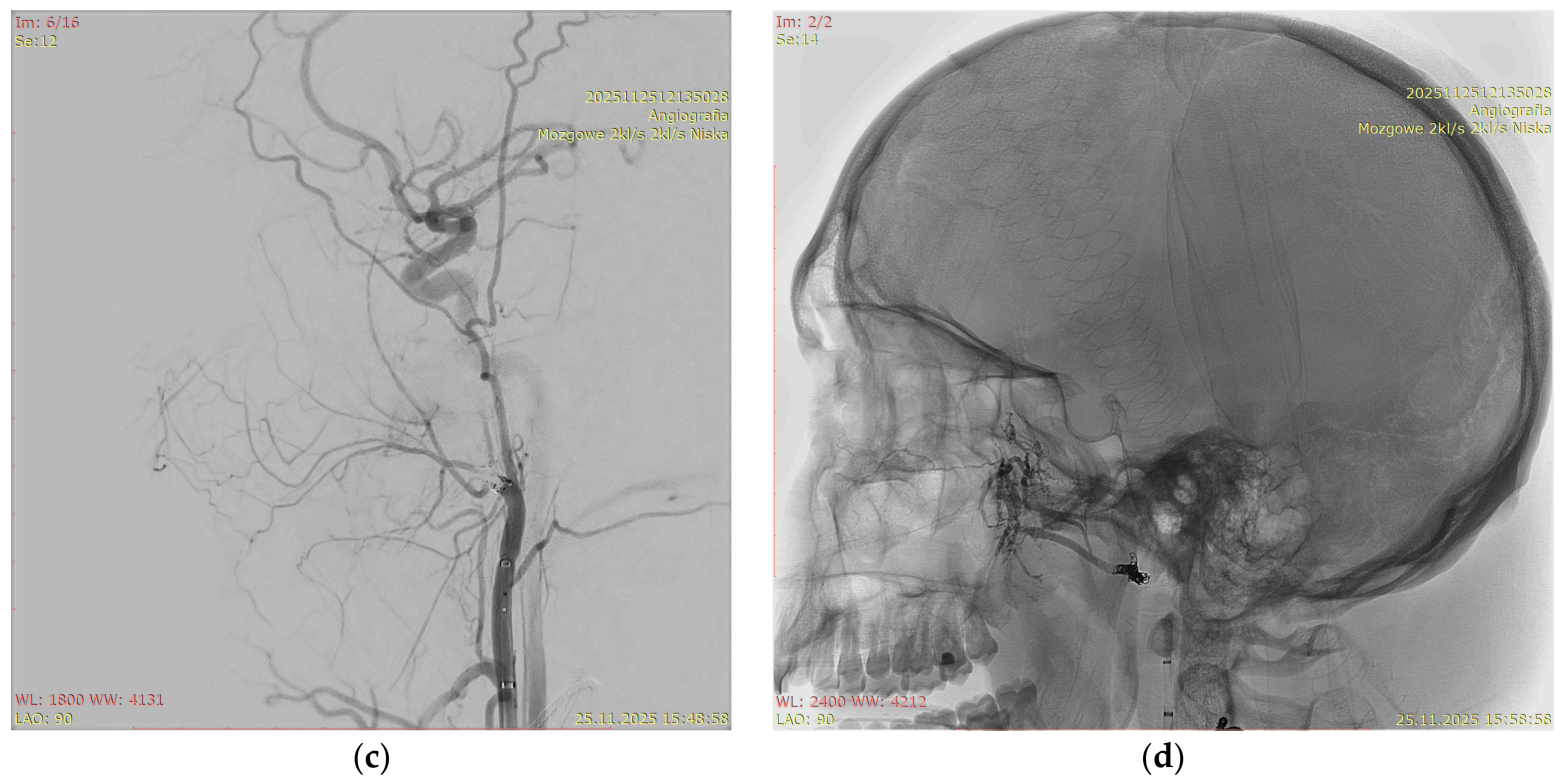
Skull-base arteriovenous malformation (AVM) in Patient Y. (**a**) Pre-treatment image of the malformation; (**b**) microarteriography of the lesion; (**c**) post-treatment vascular image; (**d**) X-ray image demonstrating the embolic material. The term “mózgowe 2 kL/s, 2 kL/s niska” is translated as “Cerebral 2 fps, 2 fps Low”.

The illustrative component of the study comprised two representative clinical cases. Figure 1 and Figure 2 present Patient X, who had a left-sided tentorial dAVF. Figure 3 and Figure 4 show Patient Y with a skull-base AVM.

### 2.1. Angiography Suite

**Figure 5:**
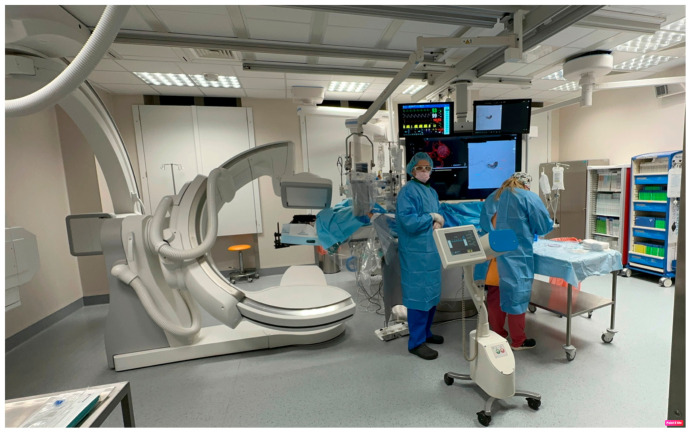
An aneurysm embolization procedure employing the Philips Azurion biplane angiography unit (W7/B20) equipped with a wide-format display monitor.

All interventions were conducted in a dedicated angiography suite equipped with a Philips Azurion ClarityIQ biplane w7 b20 system (Philips Medical Systems, Best, The Netherlands), featuring a 30 × 30 cm flat-panel detector, three-dimensional (3D) imaging capability, and advanced visualization tools (Figure 5). This system supports both conventional image display and wide-screen presentation. In selected single-plane procedures, the wide-screen layout reduced the need for additional magnification, potentially decreasing radiation exposure for both patients and staff.

The Azurion system employs automatic beam filtration, dynamically inserting copper-aluminum filters based on patient thickness, field size, source-to-image distance (SID), and selected exposure settings. This process eliminates the low-energy portion of the X-ray spectrum while preserving angiographic contrast and clinically adequate image quality. Additionally, the system automatically adjusts tube voltage, tube current, and frame rate.

### 2.2. Low-Dose Technique

Throughout the study period, all endovascular procedures followed a standardized workflow intended to reduce radiation exposure while preserving procedural safety and diagnostically adequate image quality [15,16,17]. This protocol used reduced acquisition rates for both fluoroscopy and digital subtraction angiography (DSA), supported by advanced angiographic technology and operator expertise in low-dose techniques. Because no contemporaneous standard-frame-rate control cohort was available, the independent contribution of the low-dose workflow to the observed dose indicators cannot be isolated in this retrospective analysis.

Operator proficiency with reduced frame rates was acquired progressively through extended clinical practice. Initial training used an angiographic system with lower technical performance than the currently installed Azurion platform, enabling adaptation to image sequences with lower temporal smoothness. This feature reflects reduced temporal resolution rather than diminished core diagnostic value. As detailed below, routine quality control assessments confirmed that the low-dose protocol remained suitable for neuroangiographic use.

The nominal acquisition settings were 2 fps for DSA and 3.125 fps for fluoroscopy. A multiphase DSA acquisition protocol was implemented, consisting of 4 s at 2 fps, 8 s at 1 fps, and a venous phase at 0.5 fps. In routine clinical practice, the actual frame rate may deviate from these nominal settings when the operator activates the automatic exposure optimization mode (LOW mode). In system dose reports, this mode was indicated by the entry: “mózgowe 2 kL/s, 2 kL/s niska” (Figure 1 and Figure 3).

Figure 1 demonstrates that the dose report for Patient X showed an actual frame rate of 1 fps for most DSA series, whereas some series were acquired at 15 fps. In contrast, the dose report for Patient Y (Figure 3) showed that most DSA series were acquired at 1 fps, with some at 0.5 fps. These variations reflect the automatic exposure optimization mechanism described above.

Under low-dose conditions, system algorithms adjust the actual frame rate and other exposure parameters based on the current imaging situation, accounting for projection geometry, filtration, system configuration, and the properties of the imaged region. Consequently, the realized frame rate could vary between consecutive series within the same procedure. These fluctuations are considered integral to the automatic dose-optimization process.

Technical parameters were retrospectively extracted from the dose reports for all included procedures to characterize the exposure settings achieved in daily practice. For the DSA series, the mean tube voltage (kV), tube current-time product per frame (mAs), filtration, and effective frame rate were calculated. The mean (±standard deviation [SD]) values for the overall cohort were as follows:

mAs: 28.54 ± 15.96 (range: 1–65),
kV: 74.37 ± 2.83 (range: 61–84),
filtration: 0.1 mm copper (Cu) + 1 mm aluminum (Al) in most cases,
fps: 2.15 ± 4.85 (range: 0.5–15).

These findings indicate that the low-dose strategy was reflected not only in nominal protocol settings but also in the actual exposure parameters used in routine clinical practice. Radiation output was further influenced by the imaging configuration required to visualize the vascular lesion adequately. A single-plane setup was employed when clinically and technically sufficient, while biplane imaging was reserved for anatomies and lesions that were more challenging, such as unfavorable locations, vessel tortuosity, or complex AVM/AVF angioarchitecture.

### 2.3. Quality Control Program

A structured quality control program was maintained in the angiography suite in accordance with manufacturer recommendations and national requirements. This program included periodic in-house testing and specialized checks at specified intervals. In addition, the system underwent scheduled service inspections to verify technical performance and to ensure that imaging parameters remained within acceptable limits for clinical neuroangiographic applications.

### 2.4. Three-Dimensional Rotational Angiography

On the Azurion platform, three-dimensional rotational angiography (3D-RA) was performed using a semi-automated workflow. Once initiated by the operator, the C-arm executed a predefined rotational sweep with automatic image acquisition, followed by on-system volumetric reconstruction. Before each procedure, the relevant program and settings for dose level, rotation range, frame rate, and contrast administration were selected.

At our institution, 3D-RA was obtained using a standardized protocol with fixed geometry and acquisition parameters: 122 images, left anterior oblique (LAO) 103°, 30 fps, and an SID of 120 cm. Although nominal settings remained constant, minor differences in DAP and K_a,r_ were observed between acquisitions. These discrepancies were attributed to variations in patient body habitus, positioning, tissue attenuation, and the behavior of the automatic exposure control system.

Among the 86 AVM and 28 AVF procedures included in the study, 3D-RA was used in seven AVM and two AVF cases. In each instance, 3D-RA was performed once per procedure. The mean DAP per single 3D-RA acquisition was 3.99 ± 0.70 Gy·cm^2^, and the mean K_a,r_ was 11.1 ± 2.2 mGy. All 3D-RA acquisitions were obtained in a single-plane configuration for both AVM and AVF procedures. A single-tube configuration was used in 12 AVM procedures and 3 AVF procedures.

### 2.5. Computed Tomography

A routine non-contrast head computed tomography (CT) scan was performed within 24 h of treatment. The purpose of this follow-up examination was early detection of post-procedural complications, including intracranial hemorrhage, cerebral infarction, or clinically significant brain edema.

The angiography suite served as a hybrid environment that supported both endovascular and selected neurosurgical procedures. This configuration permitted intra-procedural CT imaging when clinically indicated. In this series, intra-procedural CT was used in 1 of 86 AVM interventions, primarily due to ventricular drainage placement. The mean DAP per CT acquisition was 28.4 ± 0.4 Gy·cm^2^, and the mean K_a,r_ was 67.5 ± 1.3 mGy.

### 2.6. Statistical Analysis

All observations meeting the inclusion criteria were retained for analysis. Due to skewed distributions and substantial inter-procedural variability in several dose-related variables, a log10 logarithmic transformation was applied. Statistical analyses were conducted using Python 3.12 with the pandas 2.3.3 and SciPy 1.16.2 libraries. Visualizations were generated using matplotlib 3.10.3.

Continuous variables were summarized as mean ± SD with range, or as median (P50) with interquartile range (P25–P75) for non-normally distributed data. The Shapiro–Wilk test was used to assess normality. Between-group comparisons were conducted using Student’s t-test for approximately normally distributed data, the Mann–Whitney U test for independent non-parametric samples, and Wilcoxon’s signed-rank test for paired analyses when appropriate. Statistical significance was set at *p* < 0.05 with a 95% confidence level.

Local DRLs were defined as the 75th percentile (P75) of the distribution of analyzed dose indicators within each procedure category, while median values corresponded to the 50th percentile (P50). Analyses were conducted for the entire cohort and separately for AVM and AVF/dAVF subgroups. Where sample size permitted, analyses were also stratified by sex.

To address the potential influence of procedural complexity, additional multivariable linear regression analyses were performed using log10-transformed radiation metrics as dependent variables. Separate models were constructed for DAP, K_a,r_, fluoroscopy time, and the number of DSA frames. The primary independent variable was lesion type (AVF vs AVM). Covariates included age, sex, number of procedures per patient, and staged treatment.

A further model was constructed to assess whether the association between lesion type and radiation exposure was mediated by acquisition intensity. For this purpose, the number of DSA frames was added to the regression model for DAP and K_a,r_. Regression coefficients from log10-transformed models were back-transformed and interpreted as percentage differences in radiation metrics. Statistical analyses were conducted using Python 3.12 with the pandas 2.3.3 and SciPy 1.16.2 libraries. Visualizations were generated using matplotlib 3.10.3.

## 3. Results

Table 4 summarizes the descriptive statistics for the entire cohort of AVM and AVF procedures (N = 114). The parameters analyzed include median values (P50), local DRLs (P75), means and standard deviations for DAP, K_a,r_, FT, and the number of DSA frames per procedure. Overall, the DRL (P75) for endovascular treatment of all malformations was 35.7 Gy·cm^2^ for DAP, 405 mGy for K_a,r_, 27 min 53 s for FT, and 357.7 for the number of DSA frames (Table 4).

The median values (P50) for AVM procedures were 22 Gy·cm^2^ for DAP (literature range: 149.6–291.8 Gy·cm^2^), 300 mGy for K_a,r_ (literature range: 1650–3073.5 mGy), 19 min 58 s for FT (literature range: 36–63.1 min), and 265 DSA frames per procedure. These values were markedly higher than those recorded for other embolized lesions treated at the center, including coil-only aneurysm embolization [16] and stent-assisted aneurysm treatment [17] (Table 5).

A comparative analysis of all procedures showed no statistically significant differences in DAP, K_a,r_, FT, or the number of DSA frames between females (n = 63) and males (n = 51). However, comparison of the AVM and AVF subgroups revealed statistically significant differences in all analyzed parameters except FT, indicating higher radiation exposure during AVF treatment than during AVM treatment.

An additional analysis within the AVM subgroup evaluated whether single-stage AVM treatment differed from other procedures in terms of DAP, K_a,r_, FT, and the number of DSA frames. No statistically significant differences were identified between these subgroups. Similarly, no differences were observed between female and male patients within the AVM subgroup regarding radiation exposure parameters and procedural characteristics.

In multivariable analysis, AVF procedures were associated with a significantly higher number of DSA frames compared with AVM procedures (*p* < 0.05). The number of frames was strongly associated with radiation exposure (*p* < 0.05).

When the number of frames was included in the regression model, the association between AVF and DAP was attenuated and was no longer statistically significant. This finding suggests that acquisition burden may partly account for the higher radiation exposure observed in AVF procedures. However, DSA frame number should be interpreted as a practical surrogate marker of acquisition intensity and procedural complexity, rather than as an independent causal determinant of radiation exposure.

## 4. Discussion

The widespread adoption of modern angiographic technology has rendered ionizing radiation-based procedures integral to contemporary neurointerventional practice. However, these advancements are accompanied by unavoidable patient exposure to ionizing radiation, necessitating ongoing dose monitoring and optimization. Consequently, establishing local DRLs has become a critical component of quality assurance in centers that routinely perform complex endovascular procedures. In accordance with the Euratom Basic Safety Standards Directive and ICRP Publication 135, DRLs serve as a practical benchmark for determining whether radiation exposure remains within the expected range for specific procedures or whether further optimization is required [18]. Importantly, the study was not designed to determine all independent anatomical predictors of radiation exposure, but to assess clinical efficacy, nor was it designed to isolate the independent causal effect of the low-dose workflow. Its primary purpose was to establish procedure-specific local DRLs and to identify practical dose-related parameters that may support radiation monitoring and optimization in daily neurointerventional practice.

The present study evaluated radiation exposure during endovascular treatment of cerebral AVMs and AVFs, and proposed local reference values based on routinely reported procedural metrics, including DAP, K_a,r_, FT, and the number of DSA frames (Table 4). A retrospective dose audit design and DRL analysis were employed, rather than a clinical outcomes study or a controlled protocol-comparison study. Accordingly, the results should be interpreted primarily in the context of procedural radiation performance.

The present analysis yielded several key observations. First, radiation exposure during AVM and AVF embolization was substantially higher than previously reported at this center for other neuroendovascular procedures performed by the same operator, including coil-only aneurysm embolization and stent-assisted aneurysm treatment [16,17] (Table 5). Second, a comparison between the AVM and AVF subgroups revealed statistically significant differences in the analyzed parameters, except for FT, indicating higher radiation exposure during AVF treatment than during AVM treatment. Third, no statistically significant sex-related differences were observed in the overall cohort or within the AVM subgroup. Finally, within the AVM subgroup, single-stage procedures did not differ significantly from other AVM interventions with respect to the analyzed exposure parameters.

These findings should be interpreted with caution in light of the available literature. Published studies on radiation exposure during endovascular treatment of cerebral AVMs and AVFs remain limited, and direct comparisons between studies are hindered by substantial methodological heterogeneity (Table 6 and Table 7). Differences among available reports include study period, cohort size, lesion type, angiographic platform, acquisition settings, case mix, reporting conventions, and the use of additional imaging techniques. Therefore, the comparisons in Table 6 and Table 7 are descriptive and serve to contextualize the present results within the spectrum of published values, rather than to assert formal claims of superiority or inferiority.

Studies summarized in Table 6 generally report high exposure levels during AVM embolization, reflecting the inherent complexity of these procedures. In the present cohort, typical exposure values for AVM treatment were lower than those reported in many previously published series. A similar observation applies to the AVF subgroup in relation to the studies summarized in Table 7, although the number of available AVF-specific reports is smaller, limiting comparability. These differences may be attributable to variation in lesion angioarchitecture, procedural complexity, treatment strategy, operator technique, and angiographic equipment. Additional factors may include differences in the proportion of highly complex cases, the number of required acquisitions, and the use of single-plane versus biplane imaging during selected procedural stages.

The relatively low values observed in the present cohort, compared with many published reports, were obtained within a standardized low-dose workflow and a highly consistent procedural environment. Several factors may have contributed to these findings, including reduced frame-rate protocols, selective use of single-plane imaging, careful acquisition planning, avoidance of unnecessary DSA runs, modern angiographic equipment, and operator experience. However, because no contemporaneous control cohort treated with conventional frame-rate settings was available, the relative contribution of each factor cannot be determined. Therefore, the observed dose indicators should be interpreted as local dose-performance data obtained under a standardized low-dose workflow, rather than as proof that the low-dose protocol alone was responsible for the lower radiation values. This controlled setting reduces intra-study variability but limits generalizability to centers with different workflows, operator experience, case mix, or equipment configurations. Accordingly, the proposed DRLs should be regarded as local reference values that reflect a highly specialized practice environment, rather than as broader population-level benchmarks.

An important finding of the present study is that AVF treatment was associated with higher radiation exposure than AVM treatment, despite no significant difference in FT. This suggests that FT alone does not adequately reflect the total radiation burden of these procedures. The elevated DAP and K_a,r_ values observed in AVF interventions may result from the need for repeated angiographic acquisitions to evaluate shunt hemodynamics, venous drainage, fistulous point occlusion, and treatment completeness. Furthermore, AVF procedures may require more frequent use of multiple working projections and repeated control runs, which increases cumulative exposure independently of fluoroscopy duration. These findings underscore the importance of using multiple complementary radiation metrics, rather than relying solely on FT, when assessing procedural dose performance. The present multivariable analysis supports this interpretation by showing that the number of DSA frames is strongly associated with radiation exposure. Nevertheless, frame count should be interpreted primarily as a practical marker of acquisition burden and procedural complexity, rather than as a fully independent explanatory factor.

The current findings demonstrate that AVM and AVF embolization procedures are significantly more dose-intensive than other embolization procedures previously analyzed at this center [16,17]. This observation is clinically plausible and reflects the technical demands of vascular malformation treatment. Such procedures typically require comprehensive lesion characterization, selective and superselective catheterization, repeated roadmap guidance, multiple DSA acquisitions, and thorough post-treatment angiographic verification. Therefore, comparisons with less complex neuroendovascular procedures should be interpreted with consideration for distinct procedural objectives and associated imaging requirements.

No statistically significant sex-related differences were identified in either the overall cohort or the AVM subgroup. These findings suggest that sex is unlikely to be a primary determinant of radiation exposure in this context. Instead, dose variability is more likely driven by lesion-specific and procedure-specific factors, such as vascular anatomy, lesion architecture, number of arterial feeders, drainage pattern, accessibility, embolization strategy, and the total number of acquisitions required. Similarly, the lack of significant differences between single-stage AVM procedures and the other AVM interventions indicates that treatment staging alone does not sufficiently capture procedural complexity. This is consistent with the regression results, which suggest that acquisition intensity, reflected by the number of DSA frames, may be more informative than simple categorical descriptors such as sex or staging alone.

From a practical perspective, the present findings support the need for lesion-specific local DRLs and for shifting focus from fluoroscopy time toward acquisition-based metrics. Reducing unnecessary DSA runs appears to be the most effective and actionable strategy for dose optimization. Grouping AVM embolization, AVF embolization, aneurysm treatment, and diagnostic cerebral angiography into a single reference category is of limited value, as these procedures differ substantially in technical complexity, procedural objectives, and anticipated radiation burden. The present data support the establishment of separate local benchmarks for AVM and AVF treatment and highlight the value of center-specific dose audits that incorporate DAP, K_a,r_, FT, and acquisition-related parameters such as the number of DSA frames.

Previous studies have demonstrated that radiation exposure in neurointerventional procedures is influenced by multiple interacting factors, including patient habitus, vascular anatomy, clinical indication, procedural complexity, number of acquisitions, fluoroscopy time and DSA frame rates, detector technology, collimation, and geometric conditions such as SID. Operator-dependent factors are also important, including familiarity with angiographic system settings, avoidance of unnecessary fluoroscopy during inactive procedural phases, and selective acquisition planning based on prior CT angiography (CTA), magnetic resonance angiography (MRA), or DSA [28]. In the present cohort, the measurable procedural factor most directly linked to exposure was the number of DSA frames. Therefore, although low-dose technology and operator familiarity are relevant, the most actionable optimization target appears to be a reduction in unnecessary acquisition burden while maintaining adequate procedural visualization.

Overall, the present findings show that relatively low local dose indicators can be observed during AVM and AVF embolization within a contemporary neurointerventional workflow using a standardized low-dose strategy on a modern angiography platform. However, because this study did not include a standard-frame-rate control group, it cannot establish the independent causal effect of the low-dose workflow. The absolute radiation exposure associated with these procedures remains higher than that observed with aneurysm embolization, reflecting the greater complexity of vascular malformation treatment. Further multicenter and multi-operator studies are required to validate transferability, assess inter-center variability, and determine whether the local DRLs proposed in this study may inform broader reference standards for AVM and AVF treatment.

## 5. Limitations

Several limitations are present in this study. It was conducted as a single-center, retrospective dose audit with DRL analysis, which inherently limits external validity and precludes causal inference. Additionally, all procedures were performed by a single, highly experienced neurointerventionalist with extensive expertise in low-frame-rate imaging and dose-optimization strategies. As a result, the reproducibility of these findings in less specialized settings and among less experienced operators is uncertain.

The analysis focused exclusively on patient radiation exposure. Occupational exposure of the primary operator, assisting staff, nurses, radiographers, and anaesthesia personnel was not assessed. Therefore, the present results should not be interpreted as providing a comprehensive evaluation of radiation protection in the angiography suite. Future studies should combine patient dose indicators with operator and staff dosimetry to better characterize the two-sided nature of radiation exposure in neurointerventional practice.

A contemporaneous control group using standard frame-rate protocols was not available. Consequently, the relatively low exposure values observed in this cohort cannot be attributed exclusively to the low-dose strategy or frame-rate reduction. The low-dose configuration represented routine clinical practice on the evaluated system throughout the study period, and conventional frame-rate settings were not implemented. A prospective protocol-comparison study involving intentional dose escalation solely for research purposes was not undertaken, as this would have exposed patients to additional radiation without direct clinical benefit. Therefore, the present study should be interpreted as a local DRL and dose-performance audit, not as a controlled efficacy study of a low-dose protocol.

All procedures utilized a single angiography platform employing vendor-specific dose-reduction and dose-optimization features. This approach limits the generalizability of findings to other systems, software configurations, and institutional workflows. Therefore, the proposed DRLs should be regarded as local benchmarks requiring validation in multicenter and multi-operator settings before broader implementation.

AVM and AVF procedures are inherently heterogeneous. Variations in lesion size, anatomical location, angioarchitecture, arterial feeders, venous drainage, embolization strategy, and procedural complexity can significantly affect radiation exposure. The AVM cohort included both classical intracranial AVMs and non-classical arteriovenous shunting lesions, including skull-base, nasal cavity, orbital, spinal, syndromic, and diagnostically complex lesions. Similarly, the AVF subgroup was relatively small and anatomically diverse. In addition, certain subgroup analyses included relatively small sample sizes, potentially limiting statistical power. Consequently, these findings should be regarded as exploratory.

The retrospective dataset lacked sufficiently structured lesion-specific and procedure-specific variables to permit robust multivariable adjustment. Detailed data on lesion morphology, objective complexity metrics, anatomical dimensions, and other potential confounders were not consistently available for the entire cohort.

Therefore, the multivariable analysis was limited to available clinical and procedural variables, including age, sex, staged treatment, number of procedures per patient, and number of DSA frames.

The number of DSA frames was used as an acquisition-related procedural variable and a practical surrogate marker of acquisition intensity. However, this parameter does not fully capture all aspects of image acquisition, including projection angle, field size, collimation, magnification, detector position, source-to-image distance, or dose per frame. Moreover, DSA frame number does not replace formal lesion-complexity assessment using complete angioarchitectural classification systems, such as Spetzler–Martin grading for AVMs or Cognard/Borden classification for AVFs. Therefore, the observed association between frame number and radiation exposure should be interpreted as evidence of acquisition-related dose contribution and procedural complexity, not as a complete or independent explanation of all exposure variability.

Because the study was retrospective, it was not possible to fully separate the effects of lesion type, operator decision-making, low-frame-rate protocols, single-plane versus biplane imaging, and equipment-related dose-reduction algorithms. These factors interact in routine clinical practice and should be evaluated prospectively in future multicenter studies.

Radiation exposure assessment was limited to the endovascular procedure. Additional imaging conducted during hospitalization, including follow-up CT scans or other radiological examinations, was excluded from the current analysis and should be evaluated separately when considering cumulative patient exposure. This limitation is particularly relevant for AVM and AVF patients, who may undergo serial DSA examinations, repeated embolization sessions, radiosurgery, and long-term angiographic follow-up over their lifetime. Therefore, the present study does not establish the cumulative radiation dose associated with the full diagnostic and therapeutic pathway of vascular malformation management. Future studies should evaluate cumulative exposure across hospitalization and long-term follow-up.

The study did not incorporate structured clinical outcome measures or a formal assessment of technical efficacy and radiation-related complications. Therefore, the findings should be interpreted primarily as dose-performance data and local reference values, rather than as evidence supporting unchanged procedural efficacy or safety.

## 6. Conclusions

Embolization of cerebral AVMs and dural AVFs is among the most radiation-intensive neuroendovascular procedures. AVF treatment was associated with higher radiation exposure than AVM treatment, despite similar fluoroscopy times. This finding indicates that FT alone does not fully reflect the total procedural radiation dose. Establishing separate local DRLs for AVM and AVF procedures may offer a valuable framework for radiation surveillance and procedure-specific dose optimization. The proposed DRLs should be interpreted as local benchmarks obtained within a standardized low-dose workflow at a single highly specialized center. Because no standard-frame-rate control cohort was available, the study cannot establish the independent causal effect of the low-dose protocol on radiation exposure.

## Figures and Tables

**Table 4:** Results for all 86 cerebral arteriovenous malformation and 28 arteriovenous fistula procedures.

Parameter	Nr	DAP (Gy·cm^2^)			*p*	K_air_ (mGy)			*p*	Number of Images			*p*	Fluoroscopy Time (s)			*p*
		P50	P75	Mean ± SD		P50	P75	Mean ± SD		P50	P75	Mean ± SD		P50	P75	Mean ± SD	
All	114	24.4	35.7	33.2 ± 49.4	-	304.5	405.5	351.7 ± 195.1	-	285	357.7	319.5 ± 149.9	-	1221	1673.3	1336.6 ± 597.1	-
Female	63	23.1	32.6	25.9 ± 12.4	*p* ≥ 0.05	297	424	34.7 ± 182.9	*p* ≥ 0.05	286	353	319.4 ± 152.1	*p* ≥ 0.05	1296	1813	1364.4 ± 604.3	*p* ≥ 0.05
Male	51	25.7	41.9	32.3 ± 21.5		338.5	400.7	372.0 ± 207.7		283.5	357.2	318.4 ± 150.1		1166	1560	1283 ± 585.9	
All AVM	86	22.0	28.9	25.9 ± 16.6	*p* < 0.05	300	400	344.7 ± 206.5	*p* < 0.05	267	310	294.2 ± 144.7	*p* < 0.05	1152	1619	128.2 ± 619.8	*p* ≥ 0.05
All AVF	28	34.8	47.3	52.5 ± 88.7		333	465	370.1 ± 0162.4		354	478	386.9 ± 144.6		1323	1820	1486.5 ± 512.1	
AVM Female	43	21.2	27.7	23.7 ± 12.5	*p* ≥ 0.05	283	419	335.9 ± 204.9	*p* ≥ 0.05	267	308.2	284.4 ± 136.2	*p* ≥ 0.05	1165	1596	1322 ± 618.8	*p* ≥ 0.05
AVM Male	43	23.2	33.7	28.1 ± 19.7		306	396	353.3 ± 210.4		267	335.5	303.6 ± 153.7		1153	1792	1273.5 ± 626.8	
One AVM procedure	35	23.3	41.2	29.9 ± 21.3	*p* ≥ 0.05	304	407	365.8 ± 232.9	*p* ≥ 0.05	267	309	309.4 ± 179.7	*p* ≥ 0.05	1152	1619	1500.8 ± 1210.3	*p* ≥ 0.05
All AVM	86	22.0	28.9	25.9 ± 16.6		300	400	344.7 ± 206.5		267	310	294.2 ± 144.7		1152	1619	1280.2 ± 619.8	

**Table 5:** Comparison of radiation exposure parameters from the present study with previously published data from the same center and operator, encompassing diagnostic cerebral angiography, coil-only aneurysm embolization, and stent-assisted aneurysm treatment.

Parameter	Nr	DAP (Gy·cm^2^)			K_air_ (mGy)			Number of Images			Fluoroscopy Time (s)		
		P50	P75	Mean ± SD	P50	P75	Mean ± SD	P50	P75	Mean ± SD	P50	P75	Mean ± SD
This study (AVM)	86	22.0	28.9	25.9 ± 16.6	300	400	344.7 ± 206.5	267	310	294.2 ± 144.7	1152	1619	128.2 ± 619.8
This study (AVF)	28	34.8	47.3	52.5 ± 88.7	333	465	370.1 ± 0162.4	354	478	386.9 ± 144.6	1323	1820	1486.5 ± 512.1
Stent-assisted coiling [17]	132	13.71	19.89	16.8 ± 10.4	219.5	332	273.27 ± 197.28	277.5	354	300.3 ±143.9	1236	1532.5	1389.2 ± 607.7
Coil only [16]	245	13.8	22.4	19.6 ± 15.5	196.0	268	226.0 ± 135.0	208	285	243.7 ± 174.0	80	1138	935.6 ± 478.1
Angiography [15]	213	7.2	13.20	23.6	50.3	92	153			301.9 ± 174.0			549 s ± 363 s

**Table 6:** Diagnostic reference levels from studies on cerebral arteriovenous malformation embolization.

Authors	Date	Number of Patients	Study	DAP (Gy·cm^2^)		K_a.r_ (mGy)		FT (min)	
				P50	P75	P50	P75	P50	P75
Acton et al. 2018 [19]		6	Local	259	310				
Ihn et al. 2021 [20]	2020–2021	78	Local	264.3	412.3	3073.5	4447.8	63.1	99
Etard et al. 2017 [21]		239	National	169.9	285	2019	3230	44.5	68
Aly et al. 2024 [22].	2019–2020	49	Local	181	247.6	1872	2384	36	48.8
Hassan et al. 2017 [23]	2017	33 (AVM I AVF)	Local	149.6		1650		57	
Ozpeynirci, Y. et al. 2022 [24]	2022	30	Local	291.8	376.2			174.1	241.8

**Table 7:** Diagnostic reference levels from studies on cerebral arteriovenous fistula embolization.

Authors	Date	Number of Patients	Study	P_KA_ (Gy·cm^2^)		K_air_ (mGy)		FT (min)	
				P50	P75	P50	P75	P50	P75
Opitz et al. 2022 [3]	2010–2020	11	Treatment	369.8	507.3	-	-	58.5	
Forbig et al. 2020 [25]	2014–2019	70		325	414	-	-	110	142
Song et al. 2021 [26]	2014–2018	50	Low dose DSA	87.9				77.4	
Kien et al. 2011 [27]					440				

## Data Availability

The original contributions presented in this study are included in the article. Further inquiries can be directed to the corresponding author.

## Abbreviations

The following abbreviations are used in this manuscript:

DAP	The dose area product, calculated as the cumulative air kerma (CAK) multiplied by the exposed area and measured in Gy·cm^2^
K_a,r_	Also known as reference point air kerma and formerly called cumulative dose or cumulative air kerma (CAK), this is the dose to air at the interventional reference point, which is 15 cm from the isocenter toward the X-ray tube. K_a,r_ is a cumulative approximation of the total radiation dose to the skin, summed over the entire procedure, and is measured in mGy
DSA	Digital subtraction angiography
FT	Fluoroscopy time
DRL	Diagnostic reference level
RTG	X-ray radiation, ionizing radiation, or Roentgen radiation
SD	Standard deviation
bAVM	Brain arteriovenous malformation
AVF	arteriovenous fistula
